# Regional Factors and Ambulatory Care–Sensitive Condition Hospitalizations in Older Japanese Adults

**DOI:** 10.1001/jamanetworkopen.2025.49457

**Published:** 2025-12-12

**Authors:** Kazuhiro Abe, Kazuki Ohashi, Shigekazu Komoto, Katsuhiko Ogasawara

**Affiliations:** 1 Department of Health Care Policy, Faculty of Medicine, Hokkaido University, Sapporo, Hokkaido, Japan; 2 Faculty of Health Sciences, Hokkaido University, Sapporo, Hokkaido, Japan; 3 Faculty of Engineering, Muroran Institute of Technology, Muroran, Hokkaido, Japan

## Abstract

**Importance:**

Hospitalization in older adults leads to a decline in daily living activities, and countries experiencing demographic aging face an increase in hospitalization rates and health care costs. Health care systems that can reduce the probability of ambulatory care–sensitive condition (ACSC) hospitalization among older populations are needed.

**Objective:**

To comprehensively assess the association between regional factors and the probability of ACSC hospitalization among older adults and the extent to which regional factors explain this probability.

**Design, Setting, and Participants:**

A retrospective cohort study in Hokkaido prefecture, Japan, focused on participants aged 65 years or older who were covered by community-based or older-age health insurance (all adults aged 72 years and older) and had at least 2 medical visits between July and December 2022. The patients were followed up until December 2023.

**Exposure:**

Explanatory variables included municipal-level factors of residence categorized according to the Andersen behavioral model, which conceptualizes health service use as a function of predisposing, enabling, and need factors. At the municipal level, 9 predisposing, 11 enabling, and 10 need factors were included.

**Main Outcomes and Measures:**

The primary outcome was the binary variable of whether an individual was hospitalized for ACSCs between January and December 2023.

**Results:**

Among the 1 272 960 participants (median [IQR] age, 78 [73-84] years; 762 118 [59.9%] women), 51 623 (4.1%) had ACSC hospitalizations: 9492 for acute conditions, 41 271 for chronic conditions, and 3779 for vaccine-preventable conditions. A multilevel logistic regression analysis with individuals for level 1 and municipalities for level 2 revealed that 6.0% of the variance was attributable to municipal factors. Lower odds of hospitalization were associated with more clinics (odds ratio [OR], 0.933; 95% CI, 0.881-0.988), long-term care rehabilitation beds (OR, 0.996; 95% CI, 0.993-0.999), and higher financial power (OR, 0.359; 95% CI, 0.236-0.548); home care support clinics showed a similar direction (OR, 0.807; 95% CI, 0.636-1.024). Higher odds were associated with more nursing home beds (OR, 1.004; 95% CI, 1.000-1.009), a higher proportion of older adults living alone (OR, 1.073; 95% CI, 1.032-1.116), and a higher income (OR, 1.0004; 95% CI, 1.0001-1.0006).

**Conclusions and Relevance:**

In this cohort study of older adults in Japan, ACSC hospitalizations were less likely in municipalities with more outpatient, in-home, and rehabilitation care resources and stronger financial capacity and more likely in those with more nursing home beds, greater social isolation, and higher income levels.

## Key Points

**Question:**

Which municipal-level factors are associated with ambulatory care–sensitive condition (ACSC) hospitalizations among older Japanese adults, and to what extent do these factors account for variations in hospitalization risk?

**Findings:**

Among over 1.27 million older adults included in this cohort study, municipalities accounted for 6.0% of variations in ACSC hospitalizations. Increased access to outpatient, home-based, and rehabilitation resources was associated with a lower risk, whereas more nursing home beds and a higher proportion of older adults living alone were associated with an increased risk.

**Meaning:**

These findings suggest that improving community health care capacity, strengthening medical long-term care coordination, and mitigating social isolation may reduce avoidable hospitalizations among older adults.

## Teaser

This cohort study evaluates the association between regional factors and the probability of ambulatory care–sensitive condition hospitalization among older adults in Hokkaido prefecture, Japan.

## Introduction

As the proportion of older adults increases, the demand for health care services, hospitalizations, and overall medical expenditures has risen.^1,2,3^ Hospitalization is associated with decreased activities of daily living and increased long-term care needs.^4^ In addition, hospitalizations for ambulatory care–sensitive conditions (ACSCs), largely preventable through effective primary care, have been linked to higher costs and functional decline.^5,6,7^ Accordingly, preventing ACSC hospitalizations, particularly among older adults, in outpatient, nursing home, and in-home care settings, is crucial. To achieve this, it is essential to establish a regional environment in which older adults receive appropriate care for ACSCs.

Regional variation in ACSC hospitalization rates has been reported in several countries.^8^ Although results differ owing to definitions of ACSCs and adjustments for confounding, ACSC hospitalization rates tend to be higher in regions with limited primary care resources, poor geographic access to primary care, and lower socioeconomic status.^6,9,10^ Furthermore, regions with higher-quality primary care (eg, continuity of care and availability of preventive services) have lower ACSC hospitalization rates.^11^ When focusing on older adults, analyses incorporating long-term care systems such as nursing home care, in-home care, and rehabilitation are needed. Furthermore, a previous systematic review^9^ reported that estimates of the association between ACSC hospitalization rates and primary care resources were often biased because of inadequate adjustment for socioeconomic and population health factors. Thus, regional factors should be comprehensively examined to obtain more accurate and unbiased estimates. In addition, to identify policy implications, it is crucial to examine the extent to which regional factors contribute to the variance in the likelihood of ACSC hospitalization among older adults.

Therefore, the objective of this study was to comprehensively examine the association between regional factors selected according to the Andersen behavioral model and the probability of ACSC hospitalization among older adults using claims data from a Japanese prefecture characterized by a super-aged population, where more than 32% of residents are aged 65 years or older. The Andersen behavioral model conceptualizes health service use as a function of predisposing, enabling, and need factors at both individual and contextual levels, providing a theoretical framework for identifying determinants of health care utilization.^12^ Furthermore, we used multilevel analysis to examine how regional factors explain variations in ACSC hospitalization probability.

## Methods

### Study Design, Participants, Setting, and Data Sources

This retrospective cohort study was conducted using medical insurance claims data (July 2022 to December 2023) from municipalities in Hokkaido, Japan. The study was approved by the Hokkaido University ethics review committee and adhered to the Declaration of Helsinki and the Strengthening the Reporting of Observational Studies in Epidemiology (STROBE) reporting guidelines for cohort studies. The requirement for informed consent was waived due to the use of deidentified data.

Participants were insured individuals aged 65 years or older with at least 2 medical visits between July and December 2022 and were followed up until December 2023. Because claims were generated only when services were used, we constructed a left-closed cohort including individuals with 2 or more outpatient visits during the prior 6 months to identify the denominator for ACSC hospitalization rates.

Japan’s universal health insurance system includes employment-based, community-based, and older-age programs (all adults aged 72 years and older). This study used community-based and older-age insurance data. Community-based programs covered self-employed workers, farmers, part-time workers, and unemployed citizens aged less than 75 years,^13^ including 63.1% of persons aged 65 to 74 years in Hokkaido as of 2022.^14^

Hokkaido is Japan’s northernmost and largest prefecture, with an area of approximately 83 450 km^2^. As of the 2020 Census, Hokkaido had 179 municipalities, a population of approximately 5.1 million, and an aging rate of 31.7% for those aged 65 years or older.^14^

### Outcome and Exposures

The primary outcome was the binary variable of whether an individual was hospitalized for ACSC (January to December 2023). In our monthly panel data, when hospitalization records appeared in 2 consecutive months, it was not possible to accurately determine whether this represented a single hospitalization lasting more than 1 month or multiple separate hospitalizations. Therefore, binary outcomes were employed. ACSCs and *International Statistical Classification of Diseases and Related Health Problems, Tenth Revision (ICD-10)* codes defined by the National Health Service in the UK were used because Japan has not yet established its own definition, and previous studies conducted in Japan have followed this approach (eTable 1 in Supplement 1).^5,13,15,16^ ACSCs were categorized as acute, including 9 conditions (eg, dehydration and gastroenteritis); chronic, including 8 conditions (eg, asthma and congestive heart failure); or vaccine-preventable, including 2 conditions (eg, pneumococcal pneumonia and influenza), according to Purdy et al.^5^

Explanatory variables were municipal factors of residence, categorized by the Andersen behavioral model (6th revision) (Box).^12^ Predisposing factors included demographics (population size, proportion of the population aged ≥65, and proportion of women aged ≥65 years), social factors (annual income per capita [per 1000 yen], proportion of primary industry workers, proportion of secondary industry workers, proportion of the population aged ≥65 years in employment, and proportion of university graduates), and belief factors (medical checkup uptake rate).

Box. **Classification of Explanatory Variables Based on the Andersen Behavioral Model (6th Revision)**

**Predisposing Factors**

**Demographic**

- Population size
- Proportion of population aged ≥65 years
- Proportion of women aged ≥65 years

**Social**

- Annual income per capita
- Proportion of primary industry workers
- Proportion of secondary industry workers
- Proportion of employed persons aged ≥65 years
- Proportion of university graduates

**Beliefs**

- Medical checkup uptake rate

**Enabling Factors**

**Financing**

- Financial power index

**Health Policy**

- Number of public health nurses

**Organization**

- Number of medical beds
- Number of clinics
- Number of physicians
- Number of long-term care health facility beds
- Number of long-term care welfare facility beds
- Number of nursing home beds
- Number of home care support hospitals
- Number of home care support clinics
- Number of home visit nursing stations

**Need Factors**

**Environmental**

- Population density
- Proportion of households with older married couples
- Proportion of households with older persons living alone

**Population Health Indices**

- Crude death rates for cancer, cardiovascular diseases, cerebrovascular diseases, pneumonia, and senility
- Mean life expectancy for men and women

Enabling factors included financing factors (financial power index; ie, the 3-year mean of base financial revenue divided by base financial demand), health policy factors (the number of local public health workers employed by municipal governments per 1000 population aged ≥65 years), and organizational factors (the number of hospital beds, clinics, physicians, long-term care health facility beds [ie, a place for rehabilitation to care recipients]), long-term care welfare facility beds (ie, a place for recipients who have difficulties living at home due to a high degree of care needed), nursing home beds (ie, facilities for recipients unable to live independently at home), home care support hospitals and clinics (ie, medical facilities that provide comprehensive home-based health care services by physicians, including around the clock medical visits, emergency hospitalization coordination, and collaboration with other health care practitioners to support patients with chronic conditions or those requiring long-term care), and home visit nursing stations (per 1000 population aged ≥65 years). Need factors included environmental factors (population density, the proportion of households with older married couples, and the proportion of households with older persons living alone) and population health indices (crude death rates of cancer, cardiovascular diseases, cerebrovascular diseases, pneumonia, and senility; ie, the 5 leading causes of death in Japan) per 1000 population, and life expectancy by sex. Data sources for all variables are summarized in eTable 2 in Supplement 1.

### Statistical Analysis

Descriptive statistics are presented as median (IQR) for each variable. Geographic variations in ACSC hospitalization rates (ie, the number of hospitalized patients divided by the number of participants) were visualized using a heat map divided into quartiles for 188 areas, including 10 wards in Sapporo, the prefectural capital. When counting hospitalizations in the 3 subcategories (acute, chronic, and vaccine-preventable), patients hospitalized multiple times in different categories were counted for each category.

Two-level multilevel logistic regression with random intercepts (level 1: individuals; level 2: municipalities) was used to examine the association between municipal factors and ACSC hospitalization. To quantify municipal-level variation, intraclass correlation coefficients (ICC), which reflect the proportion of total variance explained by municipal-level factors, and median odds ratios (OR), which express the median relative difference in odds between municipalities, were calculated.^17^ We estimated the following null models: model 1 with predisposing factors, model 2 with enabling factors, and model 3 with need factors. The percentage of proportional change in variance (PCV) was calculated.

As a sensitivity analysis, an analysis was performed for those aged 75 years or older to account for selection bias from the population coverage of the community-based health insurance claims data. Additionally, to account for the impact of the COVID-19 pandemic on the results, we analyzed ACSC hospitalizations in 2019 for those who had at least 2 visits between July and December 2018. Furthermore, we performed a multilevel analysis with each hospitalization as a binary outcome to account for the heterogeneity of the results according to the 3 subcategories (ie, acute, chronic, and vaccine-preventable conditions). All analyses were conducted using Stata MP version 16 (StataCorp LLC), with a 2-sided *P* < .05 considered significant.

## Results

This study included 1 272 960 participants (median [IQR] age, 78 [73–84] years; 762 118 [59.9%] women), of whom 51 623 (4.1%) experienced at least 1 ACSC hospitalization in 2023: 9492 admissions for acute, 41 271 for chronic, and 3779 for vaccine-preventable conditions. The Figure shows variation in ACSC hospitalization rates across municipalities (median [IQR] rate, 4.53 [3.60-6.89]); higher rates were observed in eastern and southern regions, similar to the distribution of chronic ACSC hospitalization rates. Table 1 presents a summary statistics of the municipal factors.

**Figure.**
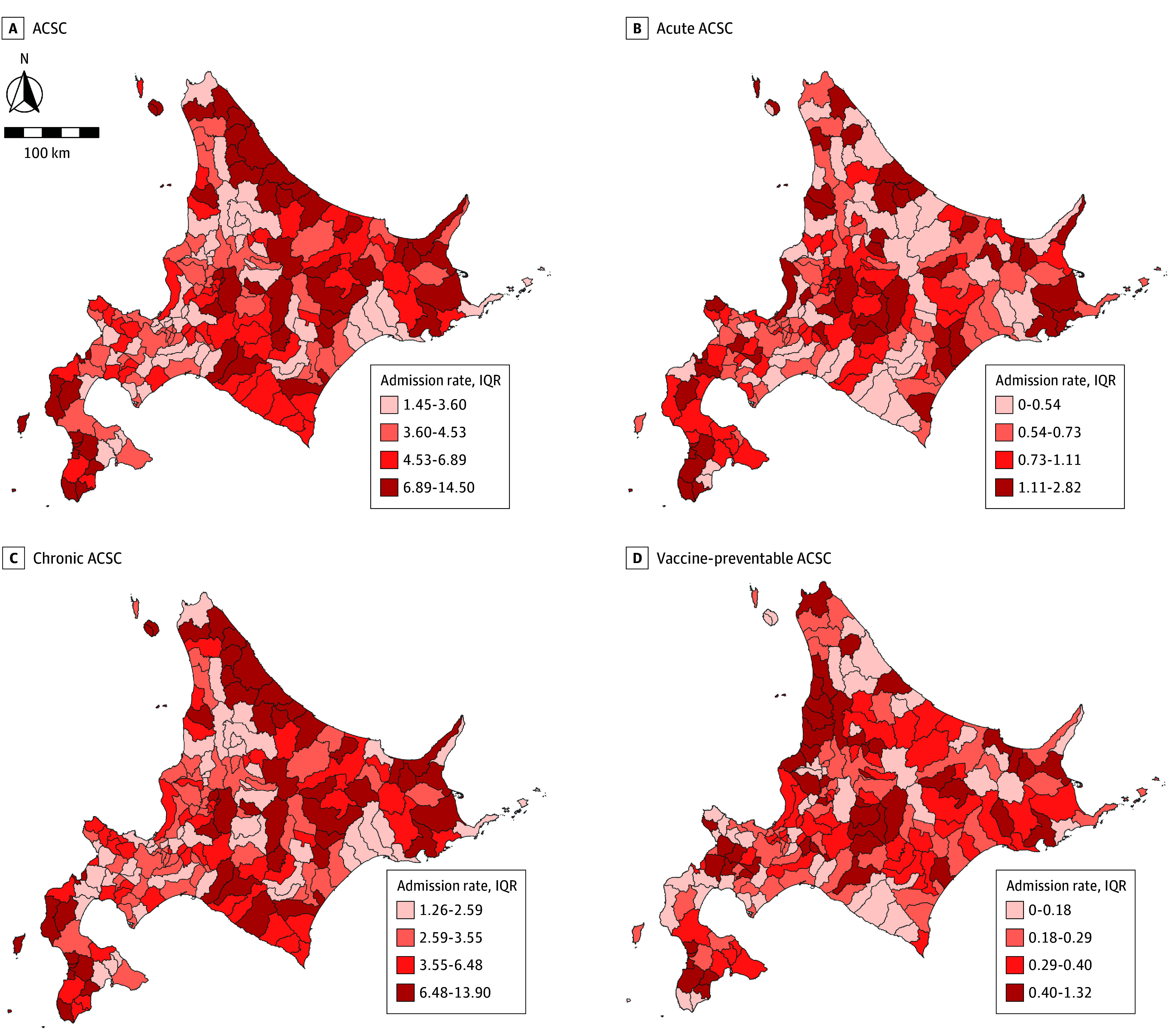
Geographical Distribution of Ambulatory Care–Sensitive Condition (ACSC) Hospitalization Rates

**Table 1.** Summary Statistics of Municipal Factors (N = 188)

Municipal factors	Median (IQR)
Predisposing	
Population size per 1000 individuals	5.8 (3.1-17.9)
Proportion of the population aged ≥ 65 y, %	37.5 (33.5-41.2)
Proportion of women aged ≥ 65 y, %	57.6 (56.5-58.5)
Annual income per capita, 1000 yen	2921.8 (2760.5-3108.6)
Proportion of primary industry workers, %	20.8 (10.2-30.9)
Proportion of secondary industry workers, %	16.1 (12.5-20.5)
Proportion of employed persons aged ≥ 65 y, %	28.0 (23.6-32.0)
Proportion of university graduates, %	9.9 (8.2-11.9)
Medical checkup uptake rate, %	33.2 (25.0-43.2)
Enabling	
Financial power index	0.25 (0.18-0.35)
No. of public health nurses^a^	2.1 (1.2-3.2)
No. of medical beds^a^	28.4 (11.6-55.1)
No. of clinics^a^	1.7 (1.1-2.3)
No. of physicians^a^	2.6 (1.5-4.4)
No. of long-term care health facility beds^a^	0 (0-14.7)
No. of long-term care welfare facility beds^a^	22.9 (13.7-38.9)
No. of nursing home beds^a^	13.3 (0.0-25.9)
No. of home care support hospitals^a^	0 (0-0)
No. of home care support clinics^a^	0 (0-0.25)
No. of home visit nursing stations^a^	0.03 (0-0.36)
Need	
Population density, persons/100 m^2^	69.8 (36.5-169.1)
Proportion of households with older married couples, %	17.1 (15.3-19.0)
Proportion of households with older persons living alone, %	16.5 (13.9-19.0)
Crude death rate of cancer	4.3 (3.5-5.3)
Crude death rate of cardiovascular diseases^b^	2.2 (1.6-3.0)
Crude death rate of cerebrovascular diseases^b^	1.1 (0.8-1.6)
Crude death rate of pneumonia^b^	0.7 (0.5-1.1)
Crude death rate of senility^b^	1.1 (0.7-1.8)
Mean life expectancy for men, y	80.9 (80.6-81.2)
Mean life expectancy for women, y	87.1 (86.9-87.3)

^a^ Per 1000 population aged 65 years or older.

^b^ Per 1000 population.

The ICC indicated that 6.0% of the variance in the probability of individual ACSC hospitalizations was attributable to municipal factors (Table 2). In addition, chronic ACSCs had the largest ICC (8.9%), followed by acute ACSCs (5.6%) and vaccine-preventable ACSCs (4.9%) (eTables 3-5 in Supplement 1). Predisposing factors were most associated with the ACSC hospitalization rate (PCV, 25.0%), followed by enabling factors (PCV, 14.3%) (Table 2).

**Table 2.** Random-Effects Parameters in the Multilevel Logistic Regression (N = 1 272 960)

Municipalities level	Null model	Model 1	Model 2	Model 3
Variance (95% CI)	0.211 (0.176-0.253)	0.158 (0.127-0.198)	0.128 (0.103-0.158)	0.110 (0.087-0.139)
Robust SE	0.020	0.018	0.014	0.013
% of Proportional change in variance, %	0	25.1	39.4	48.1
Intraclass correlation coefficient (95% CI)	0.060 (0.051-0.072)	0.046 (0.037-0.057)	0.037(0.031-0.046)	0.032 (0.026-0.041)
Median odds ratio (95% CI)	1.550 (1.492-1.616)	1.462 (1.406-1.530)	1.407 (1.358-1.461)	1.372 (1.326-1.428)

In model 3 (Table 3), lower ORs of ACSC hospitalization were associated with more clinics (OR, 0.933; 95% CI, 0.881-0.988), long-term care health facility beds (OR, 0.996; 95% CI, 0.993-0.999), and a higher financial power index (OR, 0.359; 95% CI, 0.236-0.548); home care support clinics showed a similar direction (OR, 0.807; 95% CI, 0.636-1.024) In contrast, higher odds were associated with households with older adults living alone (OR, 1.073; 95% CI, 1.032-1.116), more nursing home beds (OR, 1.004; 95% CI, 1.000-1.009), home care support hospitals, and higher income (OR, 1.0004; 95% CI, 1.0001-1.0006). In eTables 6-8 in Supplement 1, similar results were obtained when chronic ACSC hospitalization was used as the outcome: a higher acute ACSC hospitalization probability was associated with more nursing home beds (OR, 1.006; 95% CI, 1.001-1.010) and a higher crude death rate due to senility (OR, 1.110; 95% CI, 1.017-1.211) in municipalities; a higher vaccine-preventable ACSC hospitalization probability was associated with a lower financial power index of municipalities (OR, 0.295; 95% CI, 0.121-0.720). Sensitivity analyses that changed the age of the target population to 75 years or older and sensitivity analyses using prepandemic (2019) data yielded results consistent with the previously stated findings (eTables 9-12 in Supplement 1).

**Table 3.** Explanatory Variables in the Multilevel Logistic Regression (N = 1 272 960)

Municipal factors	Odds ratio (95% CI)		
	Model 1	Model 2	Model 3
Predisposing			
Population size per 1000 individuals	0.999 (0.997 to 1.000)	0.999 (0.998 to 1.000)	0.999 (0.998 to 1.000)
Proportion of the population aged ≥65 y	1.034 (1.016 to 1.052)	1.019 (1.002 to 1.036)	0.993 (0.961 to 1.027)
Proportion of women aged ≥65 y	1.013 (0.957 to 1.073)	0.995 (0.940 to 1.054)	0.958 (0.898 to 1.021)
Annual income per capita	1.0004 (1.0002 to 1.0006)	1.0004 (1.0002 to 1.0006)	1.0004 (1.0001 to 1.0006)
Proportion of primary industry workers	0.993 (0.981 to 1.005)	0.989 (0.978 to 1.000)	0.998 (0.988 to 1.009)
Proportion of secondary industry workers	1.004 (0.988 to 1.021)	1.002 (0.986 to 1.018)	1.009 (0.994 to 1.024)
Proportion of employed persons aged ≥65 y	1.018 (0.999 to 1.038)	1.010 (0.992 to 1.028)	1.008 (0.990 to 1.026)
Proportion of university graduates	1.007 (0.981 to 1.034)	1.003 (0.974 to 1.033)	1.007 (0.975 to 1.040)
Medical checkup uptake rate	0.997 (0.991 to 1.003)	0.996 (0.990 to 1.002)	0.998 (0.993 to 1.004)
Enabling			
Financial power index	NA	0.282 (0.160 to 0.497)	0.359 (0.236 to 0.548)
No. of public health nurses	NA	1.020 (0.973 to 1.068)	1.024 (0.979 to 1.070)
No. of medical beds	NA	1.000 (0.998 to 1.002)	1.001 (0.999 to 1.003)
No. of clinics	NA	0.941 (0.888 to 0.998)	0.933 (0.881 to 0.988)
No. of physicians	NA	1.013 (0.987 to 1.039)	1.012 (0.987 to 1.037)
No. of long-term care health facility beds	NA	0.997 (0.994 to 1.000)	0.996 (0.993 to 0.999)
No. of long-term care welfare facility beds	NA	1.001 (0.999 to 1.004)	1.002 (0.999 to 1.004)
No. of nursing home beds	NA	1.002 (0.998 to 1.007)	1.004 (1.000 to 1.009)
No. of home care support hospitals	NA	1.553 (0.703 to 3.431)	2.092 (0.928 to 4.713)
No. of home care support clinics	NA	0.871 (0.667 to 1.137)	0.807 (0.636 to 1.024)
No. of home visit nursing stations	NA	1.085 (0.925 to 1.272)	1.110 (0.957 to 1.287)
Need			
Population density	NA	NA	1.00000 (0.99995 to 1.00005)
Proportion of households with older married couples	NA	NA	0.988 (0.951 to 1.026)
Proportion of households with older persons living alone	NA	NA	1.073 (1.032 to 1.116)
Crude death rate of cancer	NA	NA	0.957 (0.899 to 1.019)
Crude death rate of cardiovascular diseases	NA	NA	1.005 (0.940 to 1.075)
Crude death rate of cerebrovascular diseases	NA	NA	0.971 (0.882 to 1.068)
Crude death rate of pneumonia	NA	NA	0.909 (0.824 to 1.003)
Crude death rate of senility	NA	NA	1.042 (0.976 to 1.113)
Mean life expectancy for men	NA	NA	0.999 (0.856 to 1.165)
Mean life expectancy for women	NA	NA	1.003 (0.829 to 1.213)
Constant^a^	0.001 (5.48 × 10^−5^ to 0.039)	0.015 (4.79 × 10^−4^ to 0.476)	0.108 (2.83 × 10^−9^ to 4.11 × 10^6^)

Abbreviation: NA, not applicable.

^a^ The null model for constant is 0.052 (0.049 to 0.056).

## Discussion

This cohort study comprehensively examined the association between ACSC hospitalizations among older adults and municipal factors of residence through a multilevel analysis using claims data in Japan. Among the participants, 4.1% had experienced at least 1 ACSC hospitalization. Municipal factors explained 6.0% of the variance in the probability of ACSC hospitalization among older individuals, with a greater association with chronic conditions. A lower probability of ACSC hospitalization was associated with more clinics, long-term care health facility beds, home care support clinics, and a higher financial power index in the municipality. Conversely, a higher probability was associated with living alone, more nursing home beds, and higher income.

A study conducted in suburban areas around Tokyo found that 0.7% of individuals aged 50 to 74 years experienced ACSC hospitalization annually.^15^ Our finding (4.1%) suggests that ACSC hospitalization rates vary considerably across regions and age groups within Japan. Accordingly, it is necessary to examine regional variations in hospitalization rates across Japan using a consistent ACSC definition. In addition, although direct comparisons are difficult because of differences in health care systems, previous reports indicate that approximately 10.4% of individuals aged 62 to 82 years in the UK and 6.9% of those aged 65 years or older in Australia experienced ACSC hospitalization.^18,19^ Based on these findings, the ACSC hospitalization rate among older adults in our study region did not appear to be excessively high.

Considering that ACSCs are defined as diseases and conditions for which hospitalization can be prevented with appropriate outpatient care, it is reasonable to expect that the ACSC hospitalizations would be lower among older adults in municipalities with more clinics, home care support clinics, and long-term care health facility beds per population. Our results align with those of prior studies^6,9,15^ demonstrating fewer ACSC hospitalizations in regions with better clinic access. In addition, a Canadian study^20^ reported that the First Nations and Inuit Home and Community Care Program was associated with lower ACSC hospitalization rates and that the impact was particularly notable in areas with limited primary health care resources. Another study of patients receiving home medical care in Japan reported that advance care planning was associated with a reduced rate of emergency transport for ACSCs.^21^ This study suggests that providing rehabilitation and ensuring access to clinics and home care services are essential to prevent ACSC hospitalizations among older adults.

A higher proportion of households with older individuals living alone was associated with a higher ACSC hospitalization rate, potentially reflecting barriers to accessing outpatient care. In other words, older adults living alone may face barriers in accessing outpatient care regularly or urgently without assistance or transportation. Additionally, nursing homes accommodating older residents unable to live independently and at a high risk of hospitalization may contribute to a higher probability of hospitalization owing to closer observation by professional caregivers. A study^22^ in Korea among older adults with dementia found that the risk of potentially avoidable hospitalization for acute diseases was approximately twice as high in the nursing home use group compared with the home service use group. In the present study, a similar trend was observed not only among patients with dementia and acute conditions but also in the general older population and chronic ACSCs.

Several possible mechanisms explain the association between a lower municipal financial power index and a higher ACSC hospitalization probability. For example, municipalities with worse financial conditions may have reduced the capacity of public medical facilities and community health services,^23^ resulting in a higher probability of ACSC admission among older adults. Conversely, a higher likelihood of ACSC hospitalization among older adults may necessitate maintaining public medical institutions that are not commensurate with the size of the municipality, and the cost of maintaining these institutions may strain municipal finances.^24^

Unexpectedly, more home care support hospitals were associated with a higher probability of ACSC hospitalization than home care support clinics. In Japan, home care support hospitals are certified in locations with no clinics within a 4-km radius, which may reflect the paucity of outpatient medical resources to a certain degree.^25^ In addition, as several previous studies have noted, hospitals may be more likely to admit patients than clinics owing to the availability of beds.^26,27,28^ Through these mechanisms, home care support hospitals may show an inverse association with ACSC hospitalizations compared with home care support clinics.

Contrary to previous studies,^10^ our study found that the probability of ACSC hospitalization was slightly higher in areas with higher income. Likewise, a study in Sweden reported minor differences in hospitalization rates among older adults based on income in the region.^29^ The mean annual income at the municipal level may not reflect the income of older adults, as most of our cohort had already retired from their jobs and were living on similar pension amounts. Furthermore, the working-age population in higher-income areas may present challenges in providing care to older adults with ACSCs in their homes.

The association between areas with high crude death rates due to senility and a high probability of acute ACSC hospitalization may be attributed to differences in the identifiability of acute ACSC. In other words, to diagnose senility as a cause of death, a patient must be continuously monitored by a health care professional, with no other diagnosis of fatal illness, as well as a slow decline in condition.^30,31,32^ In addition, the patient’s and family’s agreement to advance care planning would be considered necessary for the physician to diagnose senility. Thus, in regions with high crude death rates for senility, health care practitioners may be more likely to recognize older patients as acutely ill, increasing the probability of acute ACSC hospitalization, which can be addressed through hospitalization.

Finally, municipal factors were not substantially associated with the variance in the probability of ACSC hospitalization at the individual level. They were also more greatly associated with chronic ACSC hospitalization than acute or vaccine-preventable ACSCs. As noted in previous studies, the ACSC hospitalization probability also requires consideration of individual and health care practitioner factors.^29,33^

### Implications

To reduce ACSC hospitalization among older adults, local governments should ensure adequate access to outpatient, home medical, and rehabilitation care, focusing on chronic ACSCs. Strengthening the coordination between medical and long-term care services, such as home care, day care, and long-term care facilities, is crucial for providing appropriate care for older adults living alone and nursing home residents.

Achieving these goals will require establishing a Japan-specific definition of ACSCs and considering the inclusion of the ACSC hospitalization rate as a numerical target in medical and long-term care service plans. Building on the findings of this study, further causal inference analyses should clarify how municipal factors influence ACSC hospitalization. The resulting evidence could guide the reallocation of hospitalization costs potentially saved through interventions that strengthen outpatient, home-based, and rehabilitation services and support older adults living alone.

### Limitations

This study had several limitations. First, a selection bias may exist because of the population coverage of community-based insurance and the participant inclusion criteria. However, sensitivity analysis among adults aged 75 years or older using census data (ie, older age insurance data) yielded consistent results. In addition, since our analysis included 93% of the 1 365 192 insured individuals aged 65 years or older in Hokkaido as of 2022, our participant inclusion criteria are unlikely to have substantially affected the study findings.^14^ Second, the observed associations (ie, the ORs shown in Table 3) do not imply causality.^34^ Additional causal analyses are needed before implementing municipal-level interventions. Third, our results may not apply to individuals or institutions because of ecological fallacy; future studies should assess the association between ACSC hospitalization and individual- and institution-level factors. Additionally, the UK-based ACSC definition may not accurately reflect the primary care context in Japan, underscoring the need for a consensus-based national definition.

## Conclusions

In this cohort study of older adults in Japan, the probability of ACSC hospitalization was lower in municipalities with a greater number of clinics, home care support clinics, long-term care health facility beds (ie, rehabilitation beds), and a higher financial power index. Conversely, it was higher in municipalities with more nursing home beds, a greater proportion of households with older persons living alone, and a higher annual income.

## Notes

Supplement 1. eTable 1. ICD-10 Codes and Categories of Ambulatory Care Sensitive Conditions eTable 2. Summary of Data Sources for Each Variable eTable 3. Random-Effects Parameters in the Multilevel Logistic Regression for Acute ACSC Hospitalizations eTable 4. Random-Effects Parameters in the Multilevel Logistic Regression for Chronic ACSC Hospitalizations eTable 5. Random-Effects Parameters in the Multilevel Logistic Regression for Vaccine-Preventable ACSC Hospitalizations eTable 6. Odds Ratios (95% CI) of Explanatory Variables in the Multilevel Logistic Regression for Acute ACSC Hospitalizations eTable 7. Odds Ratios (95% CI) of Explanatory Variables in the Multilevel Logistic Regression for Chronic ACSC Hospitalizations eTable 8. Odds Ratios (95% CI) of Explanatory Variables in the Multilevel Logistic Regression for Vaccine–Preventable ACSC Hospitalizations eTable 9. Random-Effects Parameters in the Multilevel Logistic Regression for Population Aged 75 Years and Older eTable 10. Odds Ratios (95% CI) of Explanatory Variables in the Multilevel Logistic Regression for Population Aged 75 Years and Older eTable 11. Random-Effects Parameters in the Multilevel Logistic Regression for Population Aged 65 Years and Older in 2019 eTable 12. Odds Ratios (95% CI) of Explanatory Variables in the Multilevel Logistic Regression for Population Aged 65 Years and Older in 2019

Supplement 2. Data Sharing Statment

## References

[1] Khan HTA, Addo KM, Findlay H. Public health challenges and responses to the growing ageing populations. Public Health Chall. 2024;3(3):e213. doi:10.1002/puh2.213 40496520 PMC12039680

[2] Walsh B. Unplanned admissions and readmissions in older people: a review of recent evidence on identifying and managing high-risk individuals. Rev Clin Gerontol. 2014;24(3):228-237. doi:10.1017/S0959259814000082

[3] de Meijer C, Wouterse B, Polder J, Koopmanschap M. The effect of population aging on health expenditure growth: a critical review. Eur J Ageing. 2013;10(4):353-361. doi:10.1007/s10433-013-0280-x 28804308 PMC5549212

[4] Loyd C, Markland AD, Zhang Y, . Prevalence of hospital-associated disability in older adults: a meta-analysis. J Am Med Dir Assoc. 2020;21(4):455-461.e5. doi:10.1016/j.jamda.2019.09.015 31734122 PMC7469431

[5] Purdy S, Griffin T, Salisbury C, Sharp D. Ambulatory care sensitive conditions: terminology and disease coding need to be more specific to aid policy makers and clinicians. Public Health. 2009;123(2):169-173. doi:10.1016/j.puhe.2008.11.001 19144363

[6] Rosano A, Loha CA, Falvo R, . The relationship between avoidable hospitalization and accessibility to primary care: a systematic review. Eur J Public Health. 2013;23(3):356-360. doi:10.1093/eurpub/cks053 22645236

[7] Covinsky KE, Palmer RM, Fortinsky RH, . Loss of independence in activities of daily living in older adults hospitalized with medical illnesses: increased vulnerability with age. J Am Geriatr Soc. 2003;51(4):451-458. doi:10.1046/j.1532-5415.2003.51152.x 12657063

[8] Busby J, Purdy S, Hollingworth W. A systematic review of the magnitude and cause of geographic variation in unplanned hospital admission rates and length of stay for ambulatory care sensitive conditions. BMC Health Serv Res. 2015;15:324. doi:10.1186/s12913-015-0964-3 26268576 PMC4535775

[9] Gibson OR, Segal L, McDermott RA. A systematic review of evidence on the association between hospitalisation for chronic disease related ambulatory care sensitive conditions and primary health care resourcing. BMC Health Serv Res. 2013;13:336. doi:10.1186/1472-6963-13-336 23972001 PMC3765736

[10] Wallar LE, De Prophetis E, Rosella LC. Socioeconomic inequalities in hospitalizations for chronic ambulatory care sensitive conditions: a systematic review of peer-reviewed literature, 1990-2018. Int J Equity Health. 2020;19(1):60. doi:10.1186/s12939-020-01160-0 32366253 PMC7197160

[11] Caminal J, Starfield B, Sánchez E, Casanova C, Morales M. The role of primary care in preventing ambulatory care sensitive conditions. Eur J Public Health. 2004;14(3):246-251. doi:10.1093/eurpub/14.3.246 15369028

[12] Andersen RM, Davidson PL, Baumeister SE. Chapter Two: Improving Access to Care. In: Kominski GF, ed. Changing the US Health Care System: Key Issues in Health Services Policy and Management. Jossey-Bass; 2014.

[13] Iba A, Tomio J, Abe K, Sugiyama T, Kobayashi Y. Hospitalizations for ambulatory care sensitive conditions in a large city of Japan: a descriptive analysis using claims data. J Gen Intern Med. 2022;37(15):3917-3924. doi:10.1007/s11606-022-07713-z 35829872 PMC9640483

[14] National Statistics Center. e-Stat (portal site of official statistics of Japan). Accessed May 10, 2025. https://www.e-stat.go.jp/en

[15] Iba A, Tomio J, Sugiyama T, Abe K, Yamada I, Kobayashi Y. Association between spatial access and hospitalization for ambulatory care sensitive conditions: a retrospective cohort study using claims data. SSM Popul Health. 2024;25:101565. doi:10.1016/j.ssmph.2023.101565 PMC10711506 38089850

[16] Abe K, Kawachi I, Iba A, Miyawaki A. In-hospital deaths from ambulatory care–sensitive conditions before and during the COVID-19 pandemic in Japan. JAMA Netw Open. 2023;6(6):e2319583. doi:10.1001/jamanetworkopen.2023.19583 37347480 PMC10288336

[17] Larsen K, Merlo J. Appropriate assessment of neighborhood effects on individual health: integrating random and fixed effects in multilevel logistic regression. Am J Epidemiol. 2005;161(1):81-88. doi:10.1093/aje/kwi017 15615918

[18] Barker I, Steventon A, Deeny SR. Association between continuity of care in general practice and hospital admissions for ambulatory care sensitive conditions: cross sectional study of routinely collected, person level data. BMJ. 2017;356:j84. doi:10.1136/bmj.j84 28148478

[19] Australian Institute of Health Welfare. Potentially preventable hospitalisations in Australia by small geographic areas, 2020–21 to 2021–22. 2024. Accessed October 10, 2025. https://www.aihw.gov.au/reports/primary-health-care/potentially-preventable-hospitalisations-2020-22

[20] Lavoie JG, Forget EL, Dahl M, Martens PJ, O’Neil JD. Is it worthwhile to invest in home care? Healthc Policy. 2011;6(4):35-48. doi:10.12927/hcpol.2011.22395 22548097 PMC3107116

[21] Inoue Y, Nishi K, Mayumi T, Sasaki J. Factors in avoidable emergency visits for ambulatory care-sensitive conditions among older patients receiving home care in Japan: a retrospective study. Intern Med. 2022;61(2):177-183. doi:10.2169/internalmedicine.7136-21 35034933 PMC8851167

[22] Kim JH, Lee Y. Potentially avoidable hospitalization among long-term care insurance beneficiaries with dementia. Korean J Fam Med. 2020;41(5):318-324. doi:10.4082/kjfm.18.0184 32316707 PMC7509129

[23] Ando M. The effects of municipal fiscal strength on unsubsidized local public services: a double-LASSO analysis. J Soc Security Res. 2017;1(4):813-833.

[24] Ministry of Internal Affairs and Communications. White paper on local public finance. 2024. Accessed October 10, 2025. https://www.soumu.go.jp/iken/zaisei/r06data/chihouzaisei_2024_en.pdf

[25] Ministry of Health, Labour and Welfare. Overview of FY2022 revision of medical fee schedules. 2022. Accessed October 10, 2025. https://www.mhlw.go.jp/content/12400000/000920430.pdf

[26] Kim AM, Park JH, Yoon TH, Kim Y. Hospitalizations for ambulatory care sensitive conditions as an indicator of access to primary care and excess of bed supply. BMC Health Serv Res. 2019;19(1):259. doi:10.1186/s12913-019-4098-x 31029134 PMC6487016

[27] Laditka JN, Laditka SB, Probst JC. More may be better: evidence of a negative relationship between physician supply and hospitalization for ambulatory care sensitive conditions. Health Serv Res. 2005;40(4):1148-1166. doi:10.1111/j.1475-6773.2005.00403.x 16033497 PMC1361189

[28] Weeks WB, Ventelou B, Paraponaris A. Rates of admission for ambulatory care sensitive conditions in France in 2009-2010: trends, geographic variation, costs, and an international comparison. Eur J Health Econ. 2016;17(4):453-470. doi:10.1007/s10198-015-0692-y 25951924

[29] Löfqvist T, Burström B, Walander A, Ljung R. Inequalities in avoidable hospitalisation by area income and the role of individual characteristics: a population-based register study in Stockholm County, Sweden. BMJ Qual Saf. 2014;23(3):206-214. doi:10.1136/bmjqs-2012-001715 24082149

[30] Lunney JR, Lynn J, Foley DJ, Lipson S, Guralnik JM. Patterns of functional decline at the end of life. JAMA. 2003;289(18):2387-2392. doi:10.1001/jama.289.18.2387 12746362

[31] Landi F, Lattanzio F, Dell’Aquila G, . Prevalence and potentially reversible factors associated with anorexia among older nursing home residents: results from the ULISSE project. J Am Med Dir Assoc. 2013;14(2):119-124. doi:10.1016/j.jamda.2012.10.022 23218843

[32] Imanaga T, Toyama T. Survey on the diagnosis of senility as the cause of death in home medical care. J Japan Prim Care Assoc. 2018;41(4):169-175. doi:10.14442/generalist.41.169

[33] Vuik SI, Fontana G, Mayer E, Darzi A. Do hospitalisations for ambulatory care sensitive conditions reflect low access to primary care? An observational cohort study of primary care usage prior to hospitalisation. BMJ Open. 2017;7(8):e015704. doi:10.1136/bmjopen-2016-015704 28827243 PMC5724125

[34] Westreich D, Greenland S. The table 2 fallacy: presenting and interpreting confounder and modifier coefficients. Am J Epidemiol. 2013;177(4):292-298. doi:10.1093/aje/kws412 23371353 PMC3626058

