# Supplementary material for: Regional Factors and Ambulatory Care–Sensitive Condition Hospitalizations in Older Japanese Adults
Source: JAMA Netw Open. 2025 Dec 12;8(12):e2549457. doi: 10.1001/jamanetworkopen.2025.49457 (PMC12701509; doi:10.1001/jamanetworkopen.2025.49457)
Supplement: Supplement 1. — eTable 1. ICD-10 Codes and Categories of Ambulatory Care Sensitive Conditions eTable 2. Summary of Data Sources for Each Variable eTable 3. Random-Effects Parameters in the Multilevel Logistic Regression for Acute ACSC Hospitalizations eTable 4. Random-Effects Parameters in the Multilevel Logistic Regression for Chronic ACSC Hospitalizations eTable 5. Random-Effects Parameters in the Multilevel Logistic Regression for Vaccine-Preventable ACSC Hospitalizations eTable 6. Odds Ratios (95% CI) of Explanatory Variables in the Multilevel Logistic Regression for Acute ACSC Hospitalizations eTable 7. Odds Ratios (95% CI) of Explanatory Variables in the Multilevel Logistic Regression for Chronic ACSC Hospitalizations eTable 8. Odds Ratios (95% CI) of Explanatory Variables in the Multilevel Logistic Regression for Vaccine–Preventable ACSC Hospitalizations eTable 9. Random-Effects Parameters in the Multilevel Logistic Regression for Population Aged 75 Years and Older eTable 10. Odds Ratios (95% CI) of Explanatory Variables in the Multilevel Logistic Regression for Population Aged 75 Years and Older eTable 11. Random-Effects Parameters in the Multilevel Logistic Regression for Population Aged 65 Years and Older in 2019 eTable 12. Odds Ratios (95% CI) of Explanatory Variables in the Multilevel Logistic Regression for Population Aged 65 Years and Older in 2019 [file jamanetwopen-e2549457-s001.pdf]

Supplemental Online Content

Abe K, Ohashi K, Komoto S, Ogasawara K. Regional factors and ambulatory care–sensitive condition hospitalizations in older Japanese adults. *JAMA Netw Open*. 2025;8(12):e2549457. doi:10.1001/jamanetworkopen.2025.49457

eTable 1. ICD-10 Codes and Categories of Ambulatory Care Sensitive Conditions

eTable 2. Summary of Data Sources for Each Variable

eTable 3. Random-Effects Parameters in the Multilevel Logistic Regression for Acute ACSC Hospitalizations

eTable 4. Random-Effects Parameters in the Multilevel Logistic Regression for Chronic ACSC Hospitalizations

eTable 5. Random-Effects Parameters in the Multilevel Logistic Regression for Vaccine-Preventable ACSC Hospitalizations

eTable 6. Odds Ratios (95% CI) of Explanatory Variables in the Multilevel Logistic Regression for Acute ACSC Hospitalizations

eTable 7. Odds Ratios (95% CI) of Explanatory Variables in the Multilevel Logistic Regression for Chronic ACSC Hospitalizations

eTable 8. Odds Ratios (95% CI) of Explanatory Variables in the Multilevel Logistic Regression for Vaccine–Preventable ACSC Hospitalizations

eTable 9. Random-Effects Parameters in the Multilevel Logistic Regression for Population Aged 75 Years and Older

eTable 10. Odds Ratios (95% CI) of Explanatory Variables in the Multilevel Logistic Regression for Population Aged 75 Years and Older

eTable 11. Random-Effects Parameters in the Multilevel Logistic Regression for Population Aged 65 Years and Older in 2019

© 2025 Abe K et al. *JAMA Network Open*.

eTable 12. Odds Ratios (95% CI) of Explanatory Variables in the Multilevel Logistic Regression for Population Aged 65 Years and Older in 2019

This supplemental material has been provided by the authors to give readers additional information about their work.

© 2025 Abe K et al. *JAMA Network Open*.

eTable 1. ICD-10 Codes and Categories of Ambulatory Care Sensitive Conditions

ACSC category	ACSC	ICD-10 code
**Acute**	Cellulitis	L03, L04, L08.0, L08.8, L08.9, L88, L98.0
	Dehydration and gastroenteritis	E86, K52.2, K52.8, K52.9
	Dental conditions	A69.0, K02, K03, K04, K05, K06, K08, K09.8, K09.9, K12, K13
	Ear, nose, and throat infections	H66, H67, J02, J03, J06, J31.2
	Gangrene	R02
	Nutritional deficiency	E40, E41, E42, E43, E55.0, E64.3
	Pelvic inflammatory disease	N70, N73, N74
	Perforated or bleeding ulcer	K25.0–K25.2, K25.4–25.6, K26.0–K26.2, K26.4–26.6, K27.0–27.2, K27.4–K27.6, K280–K282, K284–K286
	Pyelonephritis	N10, N11, N12, N13.6
**Chronic**	Angina	I20, I24.0, I24.8, I24.9
	Asthma	J45, J46
	Chronic obstructive pulmonary disease	J20, J41, J42, J43, J44, J47
	Congestive heart failure	I11.0, I50, J81
	Convulsions and epilepsy	G40, G41, R56, O15
	Diabetes complications	E10.0–E10.8, E11.0–E11.8, E12.0–E12.8, E13.0–E13.8, E14.0–E14.8
	Hypertension	I10, I11.9
	Iron-deficiency anemia	D50.1, D50.8, D50.9
**Vaccine- preventable**	Influenza and pneumonia	J10, J11, J13, J14, J15.3, J15.4, J15.7, J15.9, J16.8, J18.1, J18.8
	Other vaccine-preventable diseases	A35, A36, A37, A80, B05, B06, B16.1, B16.9, B18.0, B18.1, B26, G00.0, M01.4

© 2025 Abe K et al. *JAMA Network Open*.

eTable 2. Summary of Data Sources for Each Variable

Variables	Data sources
**Outcome**	
Annual ACSC admission rate, %	Hokkaido community-based and late-elderly health insurance claims data
**Predisposing**	
Population size per 1000 people	Population Census in 2020
Proportion of the population ≧ 65 years of age, %	Population Census in 2020
Proportion of women ≧ 65 years of age, %	Population Census in 2020
Annual income per capita, 1,000 yen	Survey of Municipal Taxation in 2020
Proportion of primary industry workers, %	Population Census in 2020
Proportion of secondary industry workers, %	Population Census in 2020
Proportion of employed persons ≧ 65 years of age, %	Population Census in 2020
Proportion of university graduates, %	Population Census in 2020
Medical checkup uptake rate, %	Implementation status of specific health checkups and specific health guidance in 2020
**Enabling**	
Financial power index	Annual Statistics on Local Public Finance in 2020
No. of public health nurses	Survey on Public Health Nurses' Activity in 2020
No. of medical beds	Survey of Medical Institutions in 2020
No. of clinics	Survey of Medical Institutions in 2020
No. of physicians	Statistics of Physicians, Dentists and Pharmacists in 2020
No. of long-term care health facility beds	Survey of Institutions and Establishments for Long-term Care in 2020

© 2025 Abe K et al. *JAMA Network Open*.

No. of long-term care welfare facility beds	Survey of Institutions and Establishments for Long-term Care in 2020
No. of nursing home beds	Survey of Institutions and Establishments for Long-term Care in 2020
No. of home care support hospitals	Ministry of Health, Labour and Welfare (https://www.mhlw.go.jp/stf/seisakunitsuite/bunya/0000061944.html)
No. of home care support clinics	Ministry of Health, Labour and Welfare (https://www.mhlw.go.jp/stf/seisakunitsuite/bunya/0000061944.html)
No. of home visit nursing stations	Survey of Institutions and Establishments for Long-term Care in 2020
**Need**	
Population density, persons/100m^2	Population Census and Statistical reports on land areas in 2020
Proportion of households with older married couples, %	Population Census in 2020
Proportion of households with older persons living alone, %	Population Census in 2020
Crude death rate of cancer	Vital statistics and population census in 2020
Crude death rate of cardiovascular diseases	Vital statistics and population census in 2020
Crude death rate of cerebrovascular diseases	Vital statistics and population census in 2020
Crude death rate of pneumonia	Vital statistics and population census in 2020
Crude death rate of senility	Vital statistics and population census in 2020
Mean life expectancy for men, years	Life Tables in 2020
Mean life expectancy for women, years	Life Tables in 2020

© 2025 Abe K et al. *JAMA Network Open*.

eTable 3. Random-Effects Parameters in the Multilevel Logistic Regression for Acute ACSC Hospitalizations (n = 1,272,960)

Municipalities level	Null model	Model 1	Model 2	Model 3
**Variances (95% CI)**	0.193 (0.145-0.258)	0.164 (0.115-0.235)	0.147 (0.104-.0207)	0.132 (0.092-0.190)
**Robust SE**	0.028	0.030	0.026	0.024
**Percentage of proportional change in variance, %**	0	15.0	24.1	31.5
**Intraclass correlation coefficients (95% CI)**	0.056 (0.042-0.073)	0.048 (0.034-0.067)	0.043 (0.031-0.059)	0.039 (0.027-0.054)
**Median odds ratios (95% CI)**	1.521 (1.425-1.608)	1.472 (1.363-1.568)	1.441 (1.343-1.528)	1.415 (1.321-1.497)

© 2025 Abe K et al. *JAMA Network Open*.

eTable 4. Random-Effects Parameters in the Multilevel Logistic Regression for Chronic ACSC Hospitalizations (n = 1,272,960)

Municipalities level	Null model	Model 1	Model 2	Model 3
**Variances (95% CI)**	0.321 (0.273-0.377)	0.243 (0.120-0.297)	0.203 (0.167-0.246)	0.173 (0.138-0.216)
**Robust SE**	0.026	0.025	0.020	0.020
**Percentage of proportional change in variance, %**	0	24.1	36.7	46.2
**Intraclass correlation coefficients (95% CI)**	0.089 (0.077-0.103)	0.069 (0.057-0.083)	0.058 (0.048-0.070)	0.050 (0.041-0.062)
**Median odds ratios (95% CI)**	1.716 (1.642-1.789)	1.601 (1.521-1.673)	1.537 (1.471-1.598)	1.486 (1.418-1.551)

© 2025 Abe K et al. *JAMA Network Open*.

eTable 5. Random-Effects Parameters in the Multilevel Logistic Regression for Vaccine-Preventable ACSC Hospitalizations (n = 1,272,960)

Municipalities level	Null model	Model 1	Model 2	Model 3
**Variances (95% CI)**	0.170 (0.109-0.264)	0.131 (0.077-0.220)	0.120 (0.071-0.204)	0.114 (0.068-0.192)
**Robust SE**	0.038	0.035	0.032	0.030
**Percentage of proportional change in variance, %**	0	23.1	29.1	32.8
**Intraclass correlation coefficients (95% CI)**	0.049 (0.032-0.074)	0.038 (0.023-0.063)	0.035 (0.021-0.058)	0.034 (0.020-0.055)
**Median odds ratios (95% CI)**	1.482 (1.343-1.603)	1.412 (1.269-1-531)	1.392 (1.256-1.503)	1.380 (1.251-1.487)

© 2025 Abe K et al. *JAMA Network Open*.

eTable 6. Odds Ratios (95% CI) of Explanatory Variables in the Multilevel Logistic Regression for Acute ACSC Hospitalizations (n = 1,272,960)

	Null	Model 1	Model 2	Model 3
**Predisposing**				
Population size per 1000 people		1.000 (0.999 – 1.001)	1.000 (0.999 – 1.002)	1.000 (0.999 – 1.002)
Proportion of the population ≧ 65 years of age		1.011 (0.992 – 1.031)	1.000 (0.978 – 1.022)	0.990 (0.934 – 1.049)
Proportion of women ≧ 65 years of age		1.070 (1.001 – 1.143)	1.045 (0.967 – 1.130)	1.040 (0.948 – 1.140)
Annual income per capita		0. 99998 (0.99970 – 1.00027)	0.99993 (0.99963 – 1.00024)	0.99985 (0.99955 – 1.00014)
Proportion of primary industry workers		1.000 (0.984 – 1.016)	0.998 (0.982 – 1.015)	0.996 (0.980 – 1.012)
Proportion of secondary industry workers		1.007 (0.989 – 1.025)	1.008 (0.988 – 1.029)	1.010 (0.989 – 1.031)
Proportion of employed persons ≧ 65 years of age		1.022 (0.992 – 1.053)	1.020 (0.991 – 1.050)	1.019 (0.989 – 1.049)
Proportion of university graduates		1.004 (0.974 – 1.036)	0.996 (0.960 – 1.034)	0.999 (0.962 – 1.038)
Medical checkup uptake rate		1.000 (0.993 – 1.007)	0.999 (0.991 – 1.007)	0.999 (0.992 – 1.007)
**Enabling**				
Financial power index			0.454 (0.185 – 1.110)	0.410 (0.163 – 1.036)
No. of public health nurses			1.035 (0.961 – 1.114)	1.042 (0.970 – 1.119)
No. of medical beds			0.999 (0.996 – 1.003)	0.999 (0.996 – 1.003)
No. of clinics			0.994 (0.915 – 1.080)	0.995 (0.919 – 1.077)

© 2025 Abe K et al. *JAMA Network Open*.

No. of physicians	1.002 (0.975 – 1.031)	1.000 (0.972 – 1.029)
No. of long-term care health facility beds	1.002 (0.998 – 1.007)	1.001 (0.997 – 1.006)
No. of long-term care welfare facility beds	0.998 (0.993 – 1.002)	0.996 (0.992 – 1.000)
No. of nursing home beds	1.005 (1.001 – 1.010)	1.006 (1.001 – 1.010)
No. of home care support hospitals	2.389 (0.644 – 8.861)	2.359 (0.849 – 6.550)
No. of home care support clinics	0.853 (0.616 – 1.182)	0.838 (0.606 – 1.160)
No. of home visit nursing stations	1.055 (0.862 – 1.292)	1.067 (0.871 – 1.307)
**Need**		
Population density		1.00002 (0.99995 – 1.00008)
Proportion of households with older married couples		1.010 (0.947 – 1.077)
Proportion of households with older persons living alone		0.973 (0.914 – 1.036)
Crude death rate of cancer		1.074 (0.988 – 1.168)
Crude death rate of cardiovascular diseases		1.101 (0.994 – 1.220)
Crude death rate of cerebrovascular diseases		1.025 (0.887 – 1.185)
Crude death rate of pneumonia		0.894 (0.773 – 1.035)
Crude death rate of senility		1.110 (1.017 – 1.211)

© 2025 Abe K et al. *JAMA Network Open*.

Mean life expectancy for men				0.945 (0.763 – 1.170)
Mean life expectancy for women				1.079 (0.831 – 1.401)
Constant	0.008 (0.007 – 0.008)	5.2×10^{-5} (8.7×10^{-7} – 0.003)	4.7×10^{-4} (3.2×10^{-6} – 0.068)	1.2×10^{-4} (1.8×10^{-14} – 7.6×10^{5})

© 2025 Abe K et al. *JAMA Network Open*.

eTable 7. Odds Ratios (95% CI) of Explanatory Variables in the Multilevel Logistic Regression for Chronic ACSC Hospitalizations (n = 1,272,960)

	Null	Model 1	Model 2	Model 3
**Predisposing**				
Population size per 1000 people		0.998 (0.997 – 1.000)	0.999 (0.997 – 1.000)	0.999 (0.998 – 1.000)
Proportion of the population ≧ 65 years of age		1.043 (1.021 – 1.066)	1.027 (1.006 – 1.049)	0.988 (0.946 – 1.031)
Proportion of women ≧ 65 years of age		1.005 (0.938 – 1.076)	0.991 (0.923 – 1.063)	0.943 (0.871 – 1.021)
Annual income per capita		1.001 (1.000 – 1.001)	1.001 (1.000 – 1.001)	1.000 (1.000 – 1.001)
Proportion of primary industry workers		0.994 (0.979 – 1.009)	0.989 (0.976 – 1.003)	1.002 (0.989 – 1.016)
Proportion of secondary industry workers		1.006 (0.987 – 1.026)	1.003 (0.984 – 1.022)	1.012 (0.994 – 1.030)
Proportion of employed persons ≧ 65 years of age		1.016 (0.991 – 1.042)	1.007 (0.983 – 1.032)	1.005 (0.980 – 1.031)
Proportion of university graduates		1.009 (0.978 – 1.042)	1.004 (0.968 – 1.042)	1.010 (0.970 – 1.051)
Medical checkup uptake rate		0.996 (0.989 – 1.002)	0.995 (0.988 – 1.002)	0.998 (0.991 – 1.004)
**Enabling**				
Financial power index			0.277 (0.129 – 0.597)	0.384 (0.223 – 0.663)
No. of public health nurses			1.030 (0.968 – 1.096)	1.035 (0.975 – 1.098)
No. of medical beds			1.000 (0.998 – 1.002)	1.001 (0.999 – 1.004)
No. of clinics			0.920 (0.848 – 0.997)	0.910 (0.842 – 0.984)
No. of physicians			1.016 (0.985 – 1.048)	1.015 (0.987 – 1.044)

© 2025 Abe K et al. *JAMA Network Open.*

No. of long-term care health facility beds	0.995 (0.991 – 0.999)	0.994 (0.991 – 0.998)
No. of long-term care welfare facility beds	1.002 (0.999 – 1.005)	1.002 (0.999 – 1.006)
No. of nursing home beds	1.002 (0.997 – 1.007)	1.005 (1.000 – 1.010)
No. of home care support hospitals	1.613 (0.649 – 4.010)	2.392 (0.929 – 6.158)
No. of home care support clinics	0.880 (0.654 – 1.184)	0.794 (0.610 – 1.032)
No. of home visit nursing stations	1.082 (0.901 – 1.300)	1.116 (0.938 – 1.327)
**Need**		
Population density		1.00000 (0.99993 – 1.00006)
Proportion of households with older married couples		0.993 (0.944 – 1.043)
Proportion of households with older persons living alone		1.106 (1.047 – 1.170)
Crude death rate of cancer		0.936 (0.864 – 1.014)
Crude death rate of cardiovascular diseases		0.990 (0.913 – 1.074)
Crude death rate of cerebrovascular diseases		0.967 (0.856 – 1.092)
Crude death rate of pneumonia		0.898 (0.797 – 1.011)
Crude death rate of senility		1.049 (0.967 – 1.138)
Mean life expectancy for men		1.007 (0.839 – 1.209)

© 2025 Abe K et al. *JAMA Network Open*.

Mean life expectancy for women				0.998 (0.797 – 1.249)
Constant	0.042 (0.039 – 0.046)	0.001 (1.9×10^{-5} – 0.050)	0.009 (1.4×10^{-4} – 0.629)	0.086 (1.2×10^{-10} – 6.3×10^{7})

© 2025 Abe K et al. *JAMA Network Open*.

eTable 8. Odds Ratios (95% CI) of Explanatory Variables in the Multilevel Logistic Regression for Vaccine–Preventable ACSC Hospitalizations (n = 1,272,960)

	Null	Model 1	Model 2	Model 3
**Predisposing**				
Population size per 1000 people		0.999 (0.998 – 1.000)	1.000 (0.998 – 1.001)	1.000 (0.998 – 1.001)
Proportion of the population ≧ 65 years of age		0.967 (0.950 – 0.985)	0.953 (0.932 – 0.974)	0.967 (0.908 – 1.029)
Proportion of women ≧ 65 years of age		1.072 (1.002 – 1.148)	1.033 (0.956 – 1.115)	1.050 (0.956 – 1.154)
Annual income per capita		1.000 (0.999 – 1.000)	1.000 (0.999 – 1.000)	1.000 (0.999 – 1.000)
Proportion of primary industry workers		1.002 (0.984 – 1.021)	1.001 (0.983 – 1.020)	0.999 (0.979 – 1.019)
Proportion of secondary industry workers		0.984 (0.963 – 1.006)	0.990 (0.967 – 1.013)	0.991 (0.968 – 1.015)
Proportion of employed persons ≧ 65 years of age		0.994 (0.960 – 1.029)	0.987 (0.953 – 1.023)	0.988 (0.955 – 1.023)
Proportion of university graduates		0.974 (0.949 – 1.001)	0.966 (0.932 – 1.001)	0.969 (0.933 – 1.007)
Medical checkup uptake rate		1.008 (1.000 – 1.015)	1.007 (0.999 – 1.015)	1.008 (1.000 – 1.015)
**Enabling**				
Financial power index			0.294 (0.125 – 0.692)	0.295 (0.121 – 0.720)
No. of public health nurses			0.977 (0.897 – 1.063)	0.981 (0.901 – 1.067)
No. of medical beds			1.001 (0.998 – 1.004)	1.001 (0.997 – 1.004)
No. of clinics			1.048 (0.943 – 1.164)	1.047 (0.947 – 1.159)
No. of physicians			1.001 (0.972 – 1.030)	1.008 (0.976 – 1.041)

© 2025 Abe K et al. *JAMA Network Open*.

No. of long-term care health facility beds	1.000 (0.994 – 1.007)	1.000 (0.993 – 1.006)
No. of long-term care welfare facility beds	0.999 (0.993 – 1.005)	0.997 (0.991 – 1.003)
No. of nursing home beds	1.002 (0.996 – 1.008)	1.000 (0.995 – 1.006)
No. of home care support hospitals	1.900 (0.478 – 7.560)	1.767 (0.423 – 7.390)
No. of home care support clinics	1.080 (0.761 – 1.532)	1.051 (0.729 – 1.515)
No. of home visit nursing stations	0.938 (0.746 – 1.179)	0.991 (0.773 – 1.270)
**Need**		
Population density		0.99997 (0.99988 – 1.00006)
Proportion of households with older married couples		0.994 (0.924 – 1.071)
Proportion of households with older persons living alone		0.961 (0.890 – 1.037)
Crude death rate of cancer		0.971 (0.863 – 1.092)
Crude death rate of cardiovascular diseases		1.052 (0.920 – 1.203)
Crude death rate of cerebrovascular diseases		1.115 (0.922 – 1.347)
Crude death rate of pneumonia		1.002 (0.829 – 1.210)
Crude death rate of senility		1.084 (0.971 – 1.209)
Mean life expectancy for men		0.904 (0.726 – 1.126)

© 2025 Abe K et al. *JAMA Network Open*.

Mean life expectancy for women				1.070 (0.837 – 1.368)
Constant	0.00297 (0.00274 – 0.00322)	0.001 (7.8×10^{-6} – 0.038)	0.013 (8.6×10^{-5} – 1.926)	0.055 (9.3×10^{-11} – 3.2×10^{7})

© 2025 Abe K et al. *JAMA Network Open*.

eTable 9. Random-Effects Parameters in the Multilevel Logistic Regression for Population Aged 75 Years and Older

Municipalities level	Null model	Model 1	Model 2	Model 3
**Variances (95% CI)**	0.239 (0.199-0.286)	0.180 (0.143-0.226)	0.147 (0.118-0.184)	0.125 (0.098-0.160)
**Robust SE**	0.022	0.021	0.017	0.016
**Percentage of proportional change in variance, %**	0	24.7	38.2	47.5
**Intraclass correlation coefficients (95% CI)**	0.068 (0.057-0.080)	0.052 (0.042-0.064)	0.043 (0.035-0.053)	0.037 (0.029-0.046)
**Median odds ratios (95% CI)**	1.593 (1.524-1.660)	1.498 (1.428-1.565)	1.442 (1.380-1.500)	1.401 (1.341-1.457)

© 2025 Abe K et al. *JAMA Network Open*.

eTable 10. Odds Ratios (95% CI) of Explanatory Variables in the Multilevel Logistic Regression for Population Aged 75 Years and Older (n = 821,157)

	Null	Model 1	Model 2	Model 3
**Predisposing**				
Population size per 1000 people		0.999 (0.998 – 1.000)	0.999 (0.998 – 1.000)	0.999 (0.998 – 1.000)
Proportion of the population ≧ 65 years of age		1.034 (1.016 – 1.053)	1.019 (1.000 – 1.037)	0.987 (0.952 – 1.023)
Proportion of women ≧ 65 years of age		1.007 (0.948 – 1.070)	0.987 (0.927 – 1.051)	0.951 (0.887 – 1.018)
Annual income per capita		1.0004 (1.0002 – 1.0007)	1.0004 (1.0002 – 1.0007)	1.0004 (1.0001 – 1.0006)
Proportion of primary industry workers		0.995 (0.982 – 1.007)	0.990 (0.979 – 1.002)	1.001 (0.989 – 1.012)
Proportion of secondary industry workers		1.003 (0.986 – 1.021)	1.002 (0.985 – 1.019)	1.009 (0.993 – 1.025)
Proportion of employed persons ≧ 65 years of age		1.018 (0.998 – 1.038)	1.011 (0.992 – 1.030)	1.009 (0.991 – 1.028)
Proportion of university graduates		1.006 (0.978 – 1.034)	1.001 (0.970 – 1.033)	1.006 (0.972 – 1.041)
Medical checkup uptake rate		0.996 (0.990 – 1.002)	0.996 (0.989 – 1.002)	0.998 (0.992 – 1.004)
**Enabling**				
Financial power index			0.270 (0.134 – 0.544)	0.352 (0.211 – 0.589)
No. of public health nurses			1.022 (0.970 – 1.076)	1.028 (0.980 – 1.078)
No. of medical beds			1.000 (0.998 – 1.002)	1.001 (0.999 – 1.003)
No. of clinics			0.928 (0.869 – 0.991)	0.918 (0.862 – 0.978)
No. of physicians			1.015 (0.986 – 1.045)	1.013 (0.986 – 1.041)

© 2025 Abe K et al. *JAMA Network Open*.

No. of long-term care health facility beds	0.997 (0.995 – 1.000)	0.997 (0.994 – 1.000)
No. of long-term care welfare facility beds	1.002 (0.999 – 1.004)	1.002 (0.999 – 1.005)
No. of nursing home beds	1.003 (0.998 – 1.007)	1.005 (1.000 – 1.009)
No. of home care support hospitals	1.633 (0.696 – 3.834)	2.316 (0.956 – 5.613)
No. of home care support clinics	0.871 (0.650 – 1.165)	0.807 (0.624 – 1.044)
No. of home visit nursing stations	1.084 (0.912 – 1.288)	1.101 (0.939 – 1.290)
**Need**		
Population density		1.000 (1.000 – 1.000)
Proportion of households with older married couples		0.992 (0.951 – 1.033)
Proportion of households with older persons living alone		1.085 (1.041 – 1.131)
Crude death rate of cancer		0.949 (0.888 – 1.014)
Crude death rate of cardiovascular diseases		1.011 (0.943 – 1.085)
Crude death rate of cerebrovascular diseases		0.962 (0.869 – 1.065)
Crude death rate of pneumonia		0.893 (0.804 – 0.993)
Crude death rate of senility		1.035 (0.965 – 1.111)
Mean life expectancy for men		1.029 (0.872 – 1.215)
Mean life expectancy for women		0.969 (0.790 – 1.188)

Constant	0.069 (0.064 – 0.074)	0.003 (7.74×10^{-5} – 0.082)	0.030 (0.001 – 1.319)	0.376 (1.85×10^{-9} – 7.64×10^{7})

© 2025 Abe K et al. *JAMA Network Open*.

eTable 11. Random-Effects Parameters in the Multilevel Logistic Regression for Population Aged 65 Years and Older in 2019

Municipalities level	Null model	Model 1	Model 2	Model 3
**Variances (95% CI)**	0.190 (0.156-0.232)	0.145 (0.118-0.179)	0.126 (0.104-0.154)	0.111 (0.090-0.136)
**Robust SE**	0.019	0.016	0.013	0.012
**Percentage of proportional change in variance, %**	0	23.6	33.6	41.8
**Intraclass correlation coefficients (95% CI)**	0.055 (0.045-0.066)	0.042 (0.035-0.052)	0.037 (0.031-0.045)	0.033 (0.027-0.040)
**Median odds ratios (95% CI)**	1.516 (1.452-1.576)	1.439 (1.380-1.492)	1.404 (1.354-1.447)	1.374 (1.325-1.420)

© 2025 Abe K et al. *JAMA Network Open*.

eTable 12. Odds Ratios (95% CI) of Explanatory Variables in the Multilevel Logistic Regression for Population Aged 65 Years and Older in 2019 (n = 1,261,942)

	Null	Model 1	Model 2	Model 3
**Predisposing**				
Population size per 1000 people		0.999 (0.998 – 1.000)	0.999 (0.998 – 1.000)	0.999 (0.998 – 1.000)
Proportion of the population ≧ 65 years of age		1.031 (1.014 – 1.048)	1.024 (1.007 – 1.042)	0.999 (0.962 – 1.038)
Proportion of women ≧ 65 years of age		1.006 (0.956 – 1.058)	0.993 (0.939 – 1.051)	0.955 (0.898 – 1.016)
Annual income per capita		1.0005 (1.0003 – 1.0007)	1.0005 (1.0003 – 1.0007)	1.0004 (1.0002 – 1.0007)
Proportion of primary industry workers		0.990 (0.978 – 1.003)	0.985 (0.973 – 0.997)	0.993 (0.981 – 1.005)
Proportion of secondary industry workers		1.002 (0.987 – 1.016)	0.998 (0.983 – 1.013)	1.004 (0.989 – 1.019)
Proportion of employed persons ≧ 65 years of age		1.019 (0.997 – 1.041)	1.018 (0.997 – 1.039)	1.017 (0.996 – 1.038)
Proportion of university graduates		0.995 (0.970 – 1.021)	0.993 (0.965 – 1.022)	0.997 (0.968 – 1.026)
Medical checkup uptake rate		0.997 (0.992 – 1.002)	0.997 (0.991 – 1.002)	0.999 (0.994 – 1.004)
**Enabling**				
Financial power index			0.493 (0.275 – 0.884)	0.611 (0.393 – 0.952)
No. of public health nurses			1.001 (0.944 – 1.061)	1.000 (0.950 – 1.053)
No. of medical beds			0.999 (0.997 – 1.001)	1.000 (0.997 – 1.002)
No. of clinics			0.927 (0.875 – 0.983)	0.920 (0.870 – 0.974)
No. of physicians			1.020 (0.993 – 1.046)	1.020 (0.995 – 1.046)

© 2025 Abe K et al. *JAMA Network Open*.

No. of long-term care health facility beds	0.998 (0.995 – 1.000)	0.997 (0.994 – 1.000)
No. of long-term care welfare facility beds	1.003 (1.001 – 1.005)	1.003 (1.000 – 1.005)
No. of nursing home beds	1.001 (0.997 – 1.005)	1.003 (0.999 – 1.007)
No. of home care support hospitals	1.535 (0.552 – 4.272)	1.868 (0.661 – 5.275)
No. of home care support clinics	0.881 (0.694 – 1.117)	0.810 (0.654 – 1.004)
No. of home visit nursing stations	1.068 (0.917 – 1.243)	1.109 (0.962 – 1.279)
**Need**		
Population density		1.000 (1.000 – 1.000)
Proportion of households with older married couples		0.991 (0.950 – 1.034)
Proportion of households with older persons living alone		1.067 (1.020 – 1.117)
Crude death rate of cancer		0.951 (0.892 – 1.015)
Crude death rate of cardiovascular diseases		1.017 (0.955 – 1.082)
Crude death rate of cerebrovascular diseases		0.974 (0.884 – 1.074)
Crude death rate of pneumonia		0.939 (0.850 – 1.037)
Crude death rate of senility		1.051 (0.987 – 1.118)
Mean life expectancy for men		0.940 (0.809 – 1.093)
Mean life expectancy for women		1.057 (0.887 – 1.259)

© 2025 Abe K et al. *JAMA Network Open*.

Constant	0.058 (0.055 – 0.062)	0.003 (1.32×10^{-4} – 0.064)	0.011 (3.35×10^{-4} – 0.385)	0.108 (1.74×10^{-8} – 6.70×10^{5})

© 2025 Abe K et al. *JAMA Network Open*.
