# Supplementary material for: Regional Factors and Ambulatory Care–Sensitive Condition Hospitalizations in Older Japanese Adults
Source: JAMA Netw Open. 2025 Dec 12;8(12):e2549457. doi: 10.1001/jamanetworkopen.2025.49457 (PMC12701509; doi:10.1001/jamanetworkopen.2025.49457)
Supplement: Supplement 2. — Data Sharing Statment [file jamanetwopen-e2549457-s002.pdf]

Data Sharing Statement

Abe. Regional Factors and Ambulatory Care–Sensitive Condition Hospitalizations in Older Japanese Adults. *JAMA Netw Open*. Published December 12, 2025.
doi:10.1001/jamanetworkopen.2025.49457

Data
Data available: No

Additional Information
Explanation for why data not available: The data used in this study were accessed with the approval of 179 municipalities in Hokkaido, Japan, and are not permitted to be shared with third parties.
